# Acute neuromechanical effects of static and PNF hamstring stretching on explosive power and balance

**DOI:** 10.3389/fphys.2026.1751808

**Published:** 2026-03-10

**Authors:** Wei-Hsun Tai, Yi-Rou Chen, Po-Ang Li, Jian-Zhi Lin, Bing-Kun Lai, Hai-Bin Yu

**Affiliations:** 1 School of Physical Education, Quanzhou Normal University, Quanzhou, China; 2 Department of Physical Education, National Taiwan University of Sport, Taichung, Taiwan

**Keywords:** balance ability, explosive, hamstring, PNF, YBT

## Abstract

**Background:**

Hamstring stretching is widely incorporated into warm-up routines, yet the acute neuromuscular consequences of different stretching modalities on flexibility, explosive performance, and balance remain unclear.

**Methods:**

Using a randomized crossover design, twenty-one healthy young adults completed three conditions: static stretching (SS), proprioceptive neuromuscular facilitation (PNF) stretching, and a no-stretch control (NS). Hamstring flexibility, vertical and broad jump performance, dynamic balance (Y-Balance Test), and static balance via center of pressure (COP) metrics were assessed immediately pre- and post-intervention.

**Results:**

PNF elicited the most favorable acute outcomes, producing significant improvements in hamstring flexibility, broad jump distance, and both static and dynamic balance compared with SS and NS (all p < 0.05). SS increased hamstring flexibility but consistently impaired static balance, reflected by larger COP area, trajectory length, and sway velocity across eyes-open and eyes-closed conditions. Vertical jump height showed no significant differences among conditions. Dynamic balance improved significantly following PNF and partially following SS.

**Conclusion:**

PNF stretching is the most effective modality for enhancing immediate flexibility, horizontal power, and postural stability, making it suitable for performance-oriented warm-ups. In contrast, static stretching may compromise neuromuscular control despite improving hamstring flexibility and is therefore better suited for non-performance or recovery contexts.

## 1 Introduction

Stretching interventions are a foundational component of exercise preparation and recovery, designed to acutely enhance muscle extensibility, joint range of motion (ROM), and neuromuscular efficiency while critically mitigating injury risk (Ingram et al., 2025). Among various modalities, static stretching (SS) involves sustained passive elongation of muscle-tendon units for a period of time, promoting viscoelastic adaptation and increased ROM through inhibition of stretch reflexes and gradual sarcomere lengthening (Guissard and Duchateau, 2004). However, acute SS has been frequently associated with transient impairments in maximal force production, explosiveness, and the rate of force development (RFD), which are attributed to reduced muscle stiffness, altered length-tension relationships, and a measurable decrease in neural drive at the muscle-tendon junction (Warneke and Lohmann, 2024). These documented acute decrements raise considerable concerns regarding SS’s functional suitability in pre-performance warm-ups for activities requiring maximal power output.

In sharp contrast, when incorporating proprioceptive neuromuscular facilitation (PNF) techniques, actively engages the neuromuscular system by employing controlled, rhythmic movements or sequences of isometric contraction followed by passive stretch (Malek et al., 2024). PNF protocols effectively elevate core and muscle temperature, enhance blood flow, and robustly potentiate alpha-motor neuron excitability, thereby improving RFD and elastic energy storage without compromising ROM (Vieira et al., 2025). Meta-analytic evidence consistently suggests that PNF elicits superior acute enhancements in explosive performance compared to SS, likely via optimized post-activation potentiation (PAP) and improved musculotendinous compliance (Aytaç and İşler, 2025). Despite the clear physiological distinctions, consensus on the optimal acute stretching modality remains elusive due to variability in protocol design, population specificity, and the scope of outcome measures.

The hamstring muscle complex, as a crucial biarticular structure spanning the hip and knee, critically modulates lower-limb kinematics and kinetics (Bramah et al., 2024). Limited hamstring extensibility functionally restricts effective hip extension during dynamic tasks, thereby diminishing stride length, vertical jump height, and the application of ground reaction force (Doyle et al., 2025). Furthermore, hamstring stiffness is an essential determinant of postural stability by regulating proprioceptive feedback from Golgi tendon organs and muscle spindles, with immediate implications for both static and dynamic balance control (Lim et al., 2014). Acute stretching-induced changes in hamstring viscoelastic properties and neural signaling may thus differentially and immediately affect power transmission, joint stability, and sensorimotor integration—the fundamental determinants of functional performance and injury susceptibility.

Although extensive literature exists on isolated stretching mechanics, few high-quality studies concurrently evaluate these multiple, functionally-integrated performance domains under controlled acute conditions (Yan et al., 2025). Prior investigations often focus narrowly on isolated outcomes such as ROM or vertical jump performance in highly athletic cohorts, neglecting a more holistic perspective that integrates responses across flexibility, explosive power, and balance in general or recreational populations (Rostami et al., 2025). Moreover, direct comparisons between a no-stretch control, SS, and the neurologically distinct PNF are scarce, limiting the evidence base for robust warm-up sequencing recommendations. This gap is particularly relevant given conflicting reports: while SS reliably improves passive ROM, its negative impact on power may acutely offset functional benefits, whereas PNF appears to confer a potentially advantageous dual benefit in mobility and force expression (Behm et al., 2016).

This study, therefore, addresses these limitations by systematically comparing the acute effects of no-stretch, static stretching, and PNF stretching hamstring protocols on lower-limb explosive power, hamstring flexibility, and balance performance. By utilizing multi-sensory testing (eyes-open vs. eyes-closed with single and double-leg stance) to provides preliminary insights into the immediate neuromuscular and biomechanical adaptations of the hamstring complex, this research aims to contributes to the understanding of surrounding stretching efficacy and directly inform evidence-based warm-up design.

## 2 Materials and methods

### 2.1 Participants and ethical approval

A total of 21 healthy, recreationally active university students were recruited (age: 20.4 ± 0.9 years; height: 1.77 ± 0.06 m; weight: 73.2 ± 8.3 kg). Inclusion criteria required participants to be free from any lower-limb or spinal injuries within the previous 6 months and to engage in moderate physical activity (defined as ≤ 3 sessions per week). An *a priori* power analysis was conducted using G*Power software (version 3.1.9.4) for a repeated-measures ANOVA (within factors). The analysis assumed a medium-to-large interaction effect size (f = 0.30), statistical power of 0.80, alpha level of 0.05, correlation among repeated measures of 0.50, and nonsphericity correction (ε) of 1. This yielded a required total sample size of 18 participants. The actual sample size of 21 exceeded this minimum requirement, thereby providing adequate statistical power to detect the hypothesized Condition × Time interaction effects. Exclusion criteria included recent surgery, acute pain, or any contraindications to stretching or explosive performance testing. This study adhered to the principles outlined in the Declaration of Helsinki and received formal approval from the Institutional Ethics Committee of Quanzhou Normal University. Prior to participation, all subjects were fully informed of the experimental procedures, potential risks, and benefits before providing written informed consent.

### 2.2 Study design and control

A randomized, within-subjects crossover design was employed to rigorously assess the acute effects of three hamstring-specific interventions: no-stretch control NS, SS, and PNF. Intervention sequences were fully randomized, and a minimum 48-h washout period was enforced between sessions to ensure complete dissipation of prior acute responses. Participants adhered to standardized pre-testing restrictions, abstaining from intense physical activity, caffeine, and alcohol for at least 24 h. All testing was conducted under tightly controlled laboratory conditions (temperature: 22 °C–24 °C; relative humidity: 50%–60%) to maximize internal validity.

### 2.3 Intervention protocols

**FIGURE 1:**
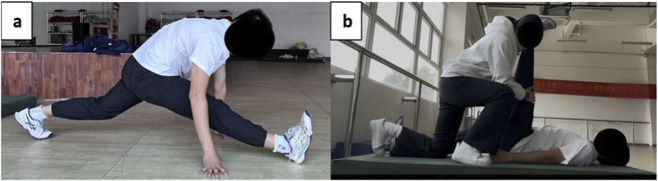
**(a)** static stretching and **(b)** PNF stretching.

All intervention sessions began with a 5-min standardized general warm-up consisting of light jogging at 6–8 km h^−1^. The hamstring specific interventions were applied unilaterally to the dominant limb by the same certified investigator using standardized verbal cues and pain anchoring on the numerical pain rating scale (NPRS) (Hrvatin and Puh, 2021). For the NS condition, participants remained seated quietly for 3 min to control for time and passive rest effects. In the SS protocol, participants performed a front back split stretch on the floor (Figure 1a) while the investigator passively flexed the hip with the knee fully extended until the participant reported mild discomfort (approximately NPRS 7/10). Each stretch was maintained for 30 s, followed by a 15-s rest, repeated three times (total stretching time: 90 s per leg). The PNF (Figure 1b) protocol followed a hold relax sequence consisting of three repeated phases: (1) a 15-s initial passive stretch to NPRS ≈ 7/10; (2) a 6-s maximal voluntary isometric hamstring contraction against resistance; and (3) immediate relaxation followed by an antagonist-assisted deeper stretch held for 15 s. Each cycle lasted 36 s and was repeated three times (total time: 108 s). Stretching intensity was standardized at a NPRS of 7/10, described to participants as ‘strong stretching but not painful.’ All interventions were administered by a single experienced examiner who underwent standardized training to ensure consistent force application and intra-operator reliability.

### 2.4 Instrumentation and data collection

**FIGURE 2:**
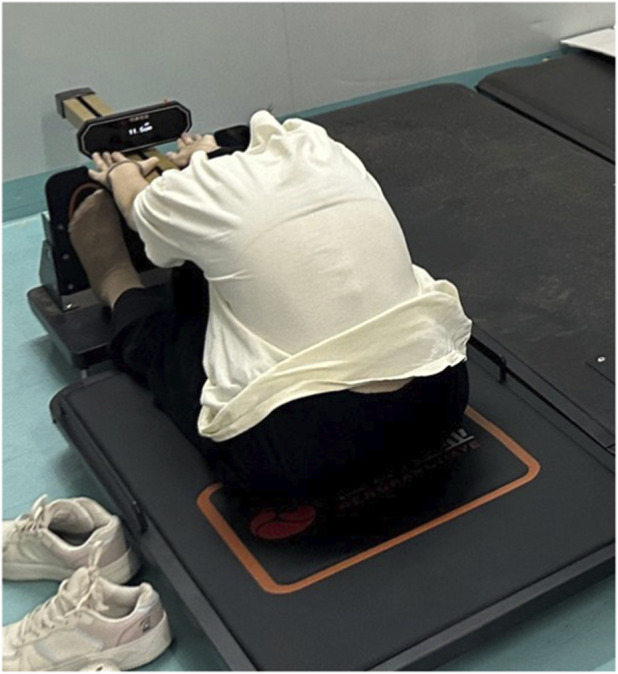
Sit and reach test (For hamstring flexibility evaluated).

**FIGURE 3:**
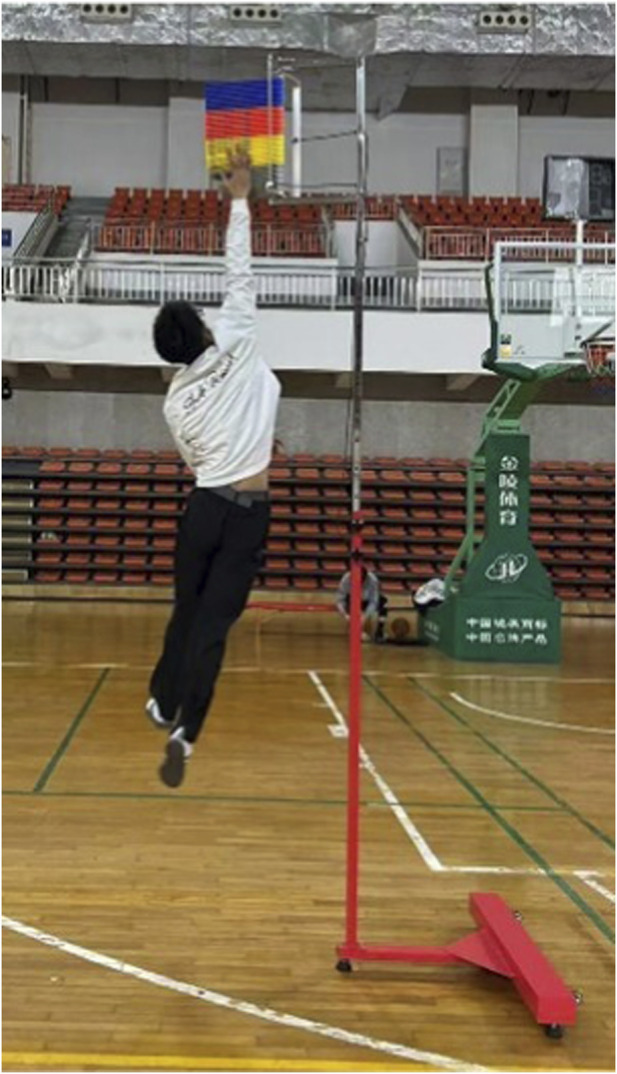
Vertical jump height evaluated.

**FIGURE 4:**
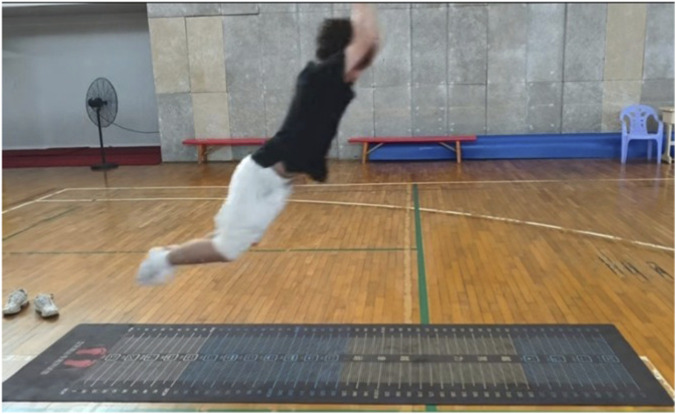
Broad jump distance evaluated.

All data were collected immediately before and within 2 min after each intervention, following a fixed, standardized testing sequence. Each assessment was performed minimum of three successful trials with standardized rest intervals between attempts, and the mean value across the three trials was used for analysis. Hamstring flexibility was evaluated using the sit and reach test, conducted with a standard sit and reach box while participants maintained full knee extension (Figure 2). The maximum forward reach distance was recorded as an indicator of hamstring flexibility. Lower-limb explosive power was assessed using two validated field-based tests: the vertical jump and the broad jump. Vertical jump height was determined as the difference between the participant’s peak and standing reach using a vertex device (Figure 3). Broad jump distance was measured on an electronic mat as the horizontal distance from the takeoff line to the heel contact point (Figure 4).

**FIGURE 5:**
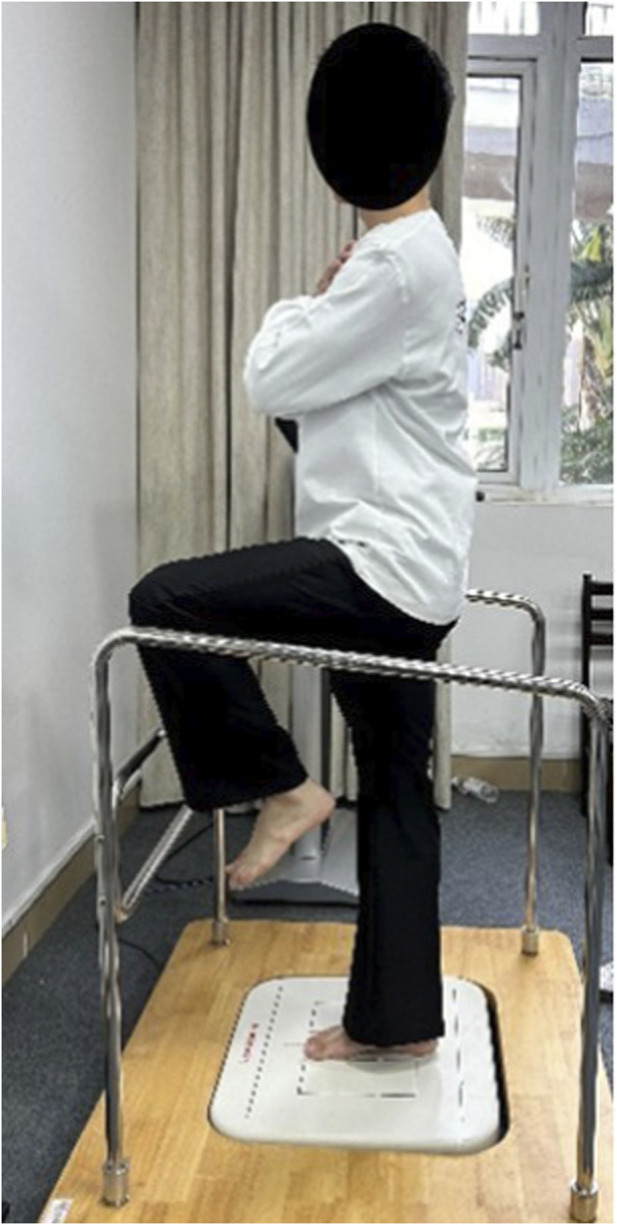
Static postural stability evaluated.

Static postural stability was evaluated using a pressure plate system (200 Hz, Acmeway Corp., Beijing, China) under four sensory and support base conditions: double-leg eyes-open (DLEO), double-leg eyes-closed (DLEC), single-leg eyes-open (SLEO), and single-leg eyes-closed (SLEC). Participants were instructed to maintain a steady upright posture with their arms crossed over the chest for the duration of each 30-s trial (Figure 5). To ensure high data fidelity, raw center of pressure (COP) trajectories were sampled at 200 Hz and subsequently smoothed using a fourth-order low-pass Butterworth filter with a 10 Hz cut-off frequency to mitigate high-frequency noise and physiological tremor artifacts. A trial was deemed valid only if the participant maintained the prescribed stance without external support, shifting the weight-bearing foot, or touching the ground with the non-weight-bearing limb. To enhance measurement reliability and minimize the influence of transient outliers, three successful trials were recorded for each condition, and the mean value was calculated for final statistical analysis. Several COP variables were collected to quantify postural control performance: Total Path Length (mm) represented the cumulative distance traveled by the COP over the 30-s interval, serving as a surrogate for total postural sway; Mean Velocity (mm/s) was derived as the path length divided by the trial duration, reflecting the average speed of postural adjustment; Sway Area (mm^2^) was calculated using the 95% confidence ellipse area encompassing the majority of COP data points to indicate the overall magnitude of the sway; and COP Ranges (mm) were determined as the maximal excursion of the COP in both the anteroposterior and mediolateral directions.

**FIGURE 6:**
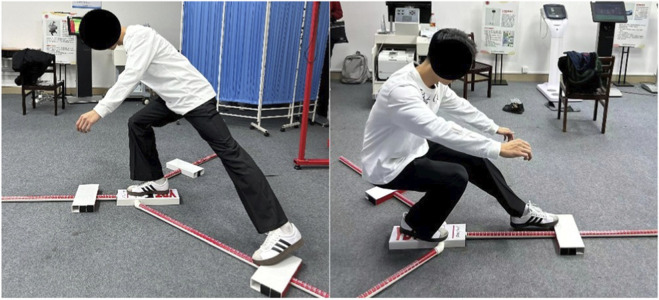
Dynamic balance evaluated (Y-Balance test).

Dynamic balance was assessed using the Y-Balance test (YBT) with a standardized testing kit (Figure 6). From a single-leg stance, maximal reach distances were recorded in the anterior, posteromedial, and posterolateral directions. Each reach distances were normalized to limb length (measured from the anterior superior iliac spine to the medial malleolus). The YBT Composite Score was calculated using the following formula (Plisky et al., 2006):

$$\text{Composite score}=\left[\frac{\left(\text{anterior}+\text{posteromedial}+\text{posterolateral}\right)}{3\times \text{limb}\;\text{length}}\right]\times 100$$

### 2.5 Statistical analysis

All statistical analyses were conducted using SPSS version 26.0 (IBM Corp., Armonk, NY, United States). Data normality was assessed using the Shapiro–Wilk test. To examine the acute effects of the three stretching modalities, a two-way (Condition × Time) repeated-measures ANOVA was performed. Sphericity was evaluated using Mauchly’s test; when violated (*p* < 0.05), degrees of freedom were adjusted with the Greenhouse-Geisser correction to ensure valid F-statistics. When a significant Condition × Time interaction or significant main effect of Time was observed, simple main effects analyses were conducted, followed by Bonferroni-adjusted pairwise comparisons to identify specific differences at post-test. Effect sizes were reported as partial eta squared (*η*_*p*_^2^) to quantify the magnitude of intervention effects. Test-retest reliability was verified using intraclass correlation coefficients (ICC) model (3, k), that employed to evaluate the inter-rater and intra-rater reliability of human-judged variables (lower-limb explosive power, hamstring flexibility, and Y-balance measurements). According to the criteria established by Koo and Li (2016), ICC values between 0.75 and 0.90 are considered good, and values greater than 0.90 are considered excellent. Statistical significance was set at *p* < 0.05.

## 3 Results

The results of the two-way repeated-measures ANOVA for all dependent variables are summarized in Table 1. Significant Condition × Time interactions were observed for hamstring flexibility, broad jump distance, and all static and dynamic balance metrics (all *p* < 0.05, *ηp* ^*2*^ range: 0.281–0.829). No significant interaction was found for vertical jump performance (*F* = 1.911, *p* = 0.162, *ηp* ^*2*^ = 0.091).

**TABLE 1:** Summary of two-way repeated measures ANOVA for all dependent variables.

Variables		*df*	F	*p*	*ηp* ^*2*^
Broad jump (cm)	Time	1, 20	7.087	0.012	0.291
	Condition	1.288, 24.468	6.695	0.011	0.261
	Condition × time	2, 40	7.413	0.004	0.281
Vertical jump (cm)	Time	1, 20	1.953	0.178	0.093
	Condition	1.300, 24.701	2.570	0.114	0.119
	Condition × time	1.212, 23.023	1.911	0.162	0.091
Hamstring flexibility (cm)	Time	1, 20	12.731	0.002	0.401
	Condition	1.488, 28.274	14.546	0.000	0.434
	Condition × time	1.399, 26.588	12.263	0.000	0.392
N-YBT-left	Time	1, 20	3.735	0.068	0.164
	Condition	2, 40	4.024	0.026	0.175
	Condition × time	2, 40	65.237	0.000	0.774
N-YBT-right	Time	1, 20	0.542	0.470	0.028
	Condition	2, 40	5.700	0.012	0.231
	Condition × time	1.341, 25.473	40.954	0.000	0.683
Range (mm)	Time	1, 20	1.890	0.184	0.086
	Condition	1.130, 22.596	24.160	0.000	0.547
	Condition × time	1.115, 22.293	22.886	0.000	0.534
DLEO-area (mm^2^)	Time	1, 20	1.493	0.236	0.069
	Condition	1.064, 21.283	23.522	0.000	0.540
	Condition × time	1.069, 21.375	23.522	0.000	0.541
DLEO-path (mm)	Time	1, 20	1.052	0.317	0.050
	Condition	2, 40	81.759	0.000	0.803
	Condition × time	2, 40	96.785	0.000	0.829
DLEO-vel (mm/s)	Time	1, 20	0.124	0.728	0.006
	Condition	1.374, 27.478	49.274	0.000	0.711
	Condition × time	1.321, 26.419	49.033	0.000	0.710
DLEC-area (mm^2^)	Time	1, 20	19.000	0.000	0.487
	Condition	2, 40	29.130	0.000	0.593
	Condition × time	2, 40	32.034	0.000	0.616
DLEC- path (mm)	Time	1, 20	2.293	0.146	0.103
	Condition	2, 40	28.423	0.000	0.587
	Condition × time	2, 40	26.567	0.000	0.571
DLEC-vel (mm/s)	Time	1, 20	2.539	0.127	0.113
	Condition	2, 40	23.255	0.000	0.538
	Condition × time	2, 40	25.668	0.000	0.562
SLEO-area (mm^2^)	Time	1, 20	0.041	0.841	0.002
	Condition	1.342, 26.839	32.634	0.000	0.620
	Condition × time	1.341, 26.823	31.838	0.000	0.614
SLEO- path (mm)	Time	1, 20	13.188	0.002	0.397
	Condition	1.244, 24.886	94.678	0.000	0.826
	Condition × time	1.256, 25.111	85.633	0.000	0.811
SLEO-vel (mm/s)	Time	1, 20	16.464	0.001	0.452
	Condition	1.290, 25.808	101.065	0.000	0.835
	Condition × time	1.206, 24.117	94.082	0.000	0.825
SLEC-area (mm^2^)	Time	1, 20	1.618	0.218	0.075
	Condition	2, 40	37.511	0.000	0.652
	Condition × time	2, 40	38.448	0.000	0.658
SLEC- path (mm)	Time	1, 20	3.445	0.078	0.147
	Condition	2, 40	29.390	0.000	0.595
	Condition × time	2, 40	29.179	0.000	0.593
SLEC-vel (mm/s)	Time	1, 20	3.076	0.095	0.133
	Condition	2, 40	29.371	0.000	0.595
	Condition × time	2, 40	26.164	0.000	0.567

Where the assumption of sphericity was violated, degrees of freedom were adjusted using the Greenhouse-Geisser correction.

Table 2 shows the simple main effect analyses following the significant interactions revealed distinct responses across conditions. For lower-limb explosive performance, PNF stretching resulted in significantly greater horizontal jump distance than both SS and NS (*F* = 8.744, *p* = 0.005, *ηp* ^*2*^ = 0.315), whereas vertical jump height did not differ significantly across conditions (*F* = 2.402, *p* = 0.131). Hamstring flexibility demonstrated that PNF producing the greatest acute improvement (15.7 ± 6.7 cm) compared to SS and NS (*F* = 13.653, *p* < 0.001, *ηp* ^*2*^ = 0.418), Dynamic balance (YBT) also showed significant differences at post-test; for the left composite score, both PNF and SS outperformed NS (*F* = 18.629, *p* < 0.001, *ηp* ^*2*^ = 0.522), while for the right composite score shows nly PNF significantly exceeded NS (*F* = 6.875, *p* = 0.008, *ηp* ^*2*^ = 0.457).

**TABLE 2:** Pre- and post-intervention performance measures and simple main effect comparisons across conditions.

Variables	NS		SS		PNF		*F*	*p*	*ηp* ^*2*^	*ICC*	*post hoc*
	Pre	Post	Pre	Post	Pre	Post					
Broad jump (cm)	245.9 ± 25.6	244.9 ± 26.6	245.3 ± 25.6	253.5 ± 19.5^#^	245.4 ± 26.2	257.4 ± 23.0^#^	8.744	0.005*	0.315	0.826	PNF > NS, SS
Vertical jump (cm)	133.4 ± 10.4	133.1 ± 10.4	132.4 ± 10.4	132.8 ± 9.3	133.2 ± 9.5	137.1 ± 15.9	2.402	0.131	0.112	0.761	
Hamstring flexibility (cm)	12.7 ± 7.1	12.7 ± 7.3	12.8 ± 7.2	14.5 ± 7.4^#^	12.8 ± 7.2	15.7 ± 6.7^#^	13.653	<0.001*	0.418	0.936	PNF > SS > NS
N-YBT-left	97 ± 7	97 ± 7	97 ± 7	101 ± 7^#^	97 ± 8	103 ± 7^#^	16.357	<0.001*	0.522	0.818	NS < SS, PNF
N-YBT-right	97 ± 8	98 ± 7	99 ± 7	100 ± 8	99 ± 7	102 ± 8^#^	12.632	<0.001*	0.457		NS < SS, PNF

*p < 0.05.

Data are presented as mean (M) ± SD; NS, none stretching; SS, static stretching; PNF, proprioceptive neuromuscular facilitation; N-YBT, normalized Y balance test.

The results of simple main effect analyses revealed significant main effects of condition for all center of pressure (COP) parameters across double-leg and single-leg stance tasks (all *p* < 0.001) in Table 3. For double-leg eyes-open (DLEO) performance, significant condition effects were observed for sway area (*F* = 23.560, *p* < 0.001, *ηp* ^*2*^ = 0.541), path length (*F* = 90.420, *p* < 0.001, *ηp* ^*2*^ = 0.819), and mean velocity (*F* = 49.504, *p* < 0.001, *ηp* ^*2*^ = 0.712). Post hoc comparisons indicated that SS resulted in significantly greater sway compared with both the NS and PNF conditions, whereas PNF demonstrated the lowest COP values (SS > NS > PNF). Under double-leg eyes-closed (DLEC) conditions, significant differences were also found for sway area (*F* = 30.734, *p* < 0.001, *ηp* ^*2*^ = 0.606), path length (*F* = 28.032, *p* < 0.001, *ηp* ^*2*^ = 0.584), and velocity (*F* = 24.954, *p* < 0.001, *ηp* ^*2*^ = 0.555). SS elicited greater postural sway compared with both NS and PNF for sway area, while PNF demonstrated significantly lower path length and velocity compared with both SS and NS.

**TABLE 3:** Pre- and post-intervention static balance performance (COP) and simple main effect comparisons across conditions.

	NS		SS		PNF		*F*	*p*	*ηp* ^*2*^	*pos hoc*
COP parameters	Pre	Post	Pre	Post	Pre	Post				
Range (mm)	12.58 ± 3.74	12.40 ± 3.72	12.54 ± 3.74	14.43 ± 4.01	12.54 ± 3.78	8.33 ± 1.70	23.649	<0.001*	0.542	PNF < NS, SS
DLEO-area (mm^2^)	425.79 ± 92.64	421.62 ± 93.46	423.46 ± 94.83	707.31 ± 409.37	424.96 ± 96.03	244.88 ± 101.83	23.560	<0.001*	0.541	SS > NS > PNF
DLEO-path (mm)	303.02 ± 36.96	300.43 ± 36.23	302.42 ± 38.19	374.04 ± 66.8	302.28 ± 36.51	211.37 ± 34.27	90.420	<0.001*	0.819	SS > NS > PNF
DLEO-vel (mm/s)	9.14 ± 1.12	9.09 ± 1.08	9.15 ± 1.12	11.79 ± 3.01	9.16 ± 1.15	6.4 ± 1.04	49.504	<0.001*	0.712	SS > NS > PNF
DLEC-area (mm^2^)	487.81 ± 176.58	479.11 ± 175.56	482.76 ± 176.39	665.96 ± 266.2	481.77 ± 174.16	472.48 ± 204.54	30.734	<0.001*	0.606	SS > NS, PNF
DLEC- path (mm)	389.17 ± 79.27	383.57 ± 79.57	385.76 ± 79.63	417.05 ± 54.68	386.10 ± 82.58	321.33 ± 79.8	28.032	<0.001*	0.584	PNF < NS, SS
DLEC-vel (mm/s)	11.86 ± 2.55	11.67 ± 2.47	11.79 ± 2.46	12.65 ± 1.62	11.79 ± 2.56	9.73 ± 2.46	24.954	<0.001*	0.555	PNF < NS, SS
SLEO-area (mm^2^)	1,244.86 ± 432.61	1,230.77 ± 434.46	1,239.32 ± 439.36	1988.52 ± 965.77	1,239.79 ± 435.57	556.87 ± 84.56	32.273	<0.001*	0.617	SS > NS > PNF
SLEO- path (mm)	586.52 ± 33.29	579.96 ± 32.09	583.49 ± 32.96	654.99 ± 118.26	583.05 ± 35.61	398.94 ± 47.08	92.495	<0.001*	0.822	SS > NS > PNF
SLEO-vel (mm/s)	27.06 ± 1.49	26.64 ± 1.34	27.00 ± 1.51	30.07 ± 5.32	26.80 ± 1.54	18.19 ± 2.15	99.918	<0.001*	0.833	SS > NS > PNF
SLEC-area (mm^2^)	2,406.80 ± 818.57	2,389.27 ± 808.8	2,405.47 ± 815.56	2,908.77 ± 813.83	2,396.14 ± 804.77	1,595.17 ± 232.64	38.288	<0.001*	0.657	SS > NS > PNF
SLEC- path (mm)	598.95 ± 126.74	593.01 ± 125.21	600.00 ± 125.57	638.03 ± 110.79	597.85 ± 127.19	501.07 ± 57.28	30.082	<0.001*	0.601	PNF < NS, SS
SLEC-vel (mm/s)	55.29 ± 11.89	57.77 ± 11.7	55.26 ± 11.87	58.77 ± 10.07	54.93 ± 11.66	46.21 ± 5.32	28.86	<0.001*	0.591	PNF < NS, SS

*p < 0.05.

Data are presented as mean (M) ± SD; COP, center of pressure; DLEO, double leg with eyes open; DLEC, double leg with eyes closed; SLEO, single leg with eyes open; SLEC, single leg with eyes closed; area, sway area; path, path length; vel, mean velocity.

In single-leg eyes-open (SLEO) stance, significant condition effects were observed for sway area (*F* = 32.273, *p* < 0.001, *ηp* ^*2*^ = 0.617), path length (*F* = 92.495, *p* < 0.001, *ηp* ^*2*^ = 0.822), and velocity (*F* = 99.918, *p* < 0.001, *ηp* ^*2*^ = 0.833). Post hoc analyses showed that SS significantly increased sway relative to NS and PNF, whereas PNF yielded the lowest COP values (SS > NS > PNF). Similarly, during single-leg eyes-closed (SLEC) stance, significant main effects of condition were detected for sway area (*F* = 38.288, *p* < 0.001, *ηp* ^*2*^ = 0.657), path length (*F* = 30.082, *p* < 0.001, *ηp* ^*2*^ = 0.601), and velocity (*F* = 28.860, *p* < 0.001, *ηp* ^*2*^ = 0.591). Static stretching resulted in greater sway area compared with both NS and PNF, while PNF exhibited significantly lower path length and velocity compared with SS and NS.

## 4 Discussion

This study systematically examined the acute effects of three distinct stretching modalities (SS, PNF, and NS) on hamstring flexibility, explosive performance, and postural balance. A key finding of our analysis was the significant Condition × Time interactions observed across nearly all performance variables. While the experimental design involved pre- and post-intervention measurements to ensure baseline equivalence, the primary focus of this research was to determine how different stretching modalities rather than the passage of time acutely influence neuromuscular outcomes. Our findings demonstrate that PNF elicited the most advantageous acute responses, producing substantial improvements in hamstring flexibility, horizontal jumping performance, and both static and dynamic balance. In contrast, SS resulted in pronounced decrements in postural stability despite improving flexibility, while exerting minimal influence on explosive performance. Collectively, these results emphasize that the specific stretching technique employed is the critical factor in determining immediate neuromuscular readiness, highlighting the importance of aligning warm-up strategies with the functional requirements of sport-specific tasks.

The superior performance observed following PNF could potentially be influenced by several complementary neuromechanical mechanisms, although these were not directly measured in the current study. Based on previous the PNF-based hold–relax sequence is hypothesized to integrates passive elongation with active isometric contraction, which may enhance stretch tolerance while preserving optimal musculotendinous stiffness (Kranjc et al., 2025). This balance between compliance and stiffness is critical for efficient force transmission and stabilization during dynamic tasks (Longo et al., 2025; Patti et al., 2025). The isometric contraction phase has been suggested in other studies to acutely elevate α-motoneuron excitability and motor unit recruitment, potentially enhancing readiness for rapid force production (Hindle et al., 2012; Reiner et al., 2021). However, in the absence of direct neurophysiological measures such as EMG or H-reflex, these mechanisms remain speculative.

Similarly, the decrements in stability observed following SS might be related to alterations in muscle spindle sensitivity or a reduction in muscle-tendon stiffness, as previously proposed in the literature (Saito and Mizuno, 2025). These neuromuscular alterations may diminish the ability to generate rapid corrective forces, particularly during single-leg stance or eyes-closed conditions where sensory integration demands are heightened (Budini et al., 2018). Additionally, increased musculotendinous compliance following SS may impair the fine-tuned modulation of joint stiffness required for maintaining postural equilibrium (Guissard and Duchateau, 2004). These mechanisms collectively explain the substantial increases in COP area, trajectory length, and velocity observed across SS conditions in the present study. However, without direct measurements of tendon stiffness or neural drive, these interpretations should be viewed with caution.

However, it is essential to acknowledge that the PNF protocol in this study involved a slightly higher total volume (108 s vs. 90 s) and higher neuromuscular intensity due to the examiner-assisted maximal isometric contractions. These contractions likely induced post-activation performance enhancement (PAPE), a phenomenon where recent voluntary contractile history acutely improves subsequent voluntary force production (Blazevich and Babault, 2019). Unlike the purely passive nature of SS, the active ‘hold’ phase in PNF may have served as a localized neuromuscular priming stimulus, increasing phosphorylation of myosin light chains and improving motor unit synchronization. Therefore, the more favorable outcomes observed in the PNF condition particularly in horizontal jumping and postural stability may stem from a combination of increased flexibility and this heightened state of neuromuscular activation, which was absent in the SS and NS protocols.

The results also align with and extend previous findings in the literature. Numerous studies have reported reductions in balance performance following SS, particularly in populations requiring precise neuromuscular control such as dancers, gymnasts, and athletes performing dynamic balance tasks (Chatzopoulos et al., 2014). Similarly, prior research has highlighted the benefits of dynamic or PNF stretching on functional performance, including enhancements in power output, proprioceptive acuity, and range of motion (Wang et al., 2024). The absence of significant effects on vertical jump height is consistent with prior work suggesting that vertical power is less sensitive to hamstring-focused stretching protocols compared with horizontal jumping tasks, which rely more heavily on posterior-chain contribution and multiplanar force application (Denerel et al., 2019). The improvements observed in YBT performance following PNF further support the notion that dynamic stretching promotes functional stability and controlled mobility, both of which contribute to multi-directional reach capability (Simões et al., 2025). In this study, stretching was applied only to the dominant limb. While the YBT reflects bilateral postural control, the observed improvements likely stem from a combination of localized flexibility gains in the dominant limb and global neuromuscular adaptations. Previous research suggests that unilateral stretching can induce cross-education effects or systemic neural drive alterations that influence contralateral or bilateral performance (Lawry-Popelka et al., 2022). Although SS contributed to YBT improvements in the left limb in this study, the lack of bilateral consistency and its detrimental effects on static balance suggest that these gains reflect increased hamstring flexibility rather than neuromuscular enhancement (Behm et al., 2023).

From a practical perspective, the findings provide clear guidance for coaches, clinicians, and practitioners designing warm-up routines. PNF appears to be the most appropriate modality when the immediate objective is to optimize explosive performance or postural control, such as during sprint starts, change-of-direction actions, balance-intensive tasks, or jumping activities (Behm et al., 2016; Behm and Chaouachi, 2011). The ability of PNF to simultaneously enhance hamstring flexibility, balance, and horizontal power makes it particularly suitable for warm-ups preceding high-demand athletic performances. In contrast, SS should be used cautiously in pre-activity settings, as the acute decrements in postural stability may increase the risk of performance impairments or even injury during tasks requiring precise neuromuscular control (Takeuchi et al., 2019). SS is therefore better positioned as a post-exercise recovery strategy or as part of long-term flexibility training rather than as an immediate preparation technique (Feng, 2024).

Several limitations should be considered. The sample was limited to healthy young adults, restricting the generalizability of the findings to older populations, clinical groups, or trained athletes. Additionally, only immediate post-intervention effects were assessed, leaving the recovery trajectory after static stretching-induced impairments in postural stability unclear; future research should examine this time course to better determine the optimal timing of flexibility exercises relative to performance tasks. Although center of pressure metrics provide reliable measures of postural control, the absence of complementary physiological data such as muscle activation, tendon stiffness, or reflex responses limits deeper mechanistic insights. Furthermore, the stretching protocols were not fully matched for total duration or intensity, meaning the apparently superior effects of PNF may be partly attributable to its neuromuscular facilitation components rather than the stretching technique itself. Future studies should therefore use strictly dose-matched designs to isolate the unique contributions of different stretching methods. Finally, hamstring flexibility was evaluated solely with the sit-and-reach test; more joint- or muscle-specific assessments could help clarify the precise locus of adaptation in subsequent work.

In summary, PNF elicited the most favorable acute effects on flexibility, explosive performance, and balance, while SS impaired static stability despite improving hamstring flexibility. These findings emphasize the need to match stretching modality with the immediate neuromuscular demands of sport or exercise. Future research should investigate longer-term adaptations, interactions with strength or plyometric training, and optimized warm-up sequencing to refine evidence-based practice.

## 5 Conclusion

This study demonstrates that stretching modality meaningfully shapes the immediate neuromuscular responses associated with physical performance. The PNF technique produced the most favorable acute outcomes, yielding concurrent improvements in hamstring flexibility, broad jump performance, and postural stability. In contrast, static stretching effectively increased hamstring flexibility but compromised static balance control and offered limited benefit for explosive performance. Accordingly, PNF appears well suited for pre-activity preparation when both mobility and neuromuscular readiness are required, whereas static stretching may be reserved for sessions prioritizing flexibility gains rather than tasks demanding immediate balance or power output. Future research should examine whether different PNF variants elicit distinct neuromechanical effects, explore interactions between stretching modalities and strength or plyometric training, and incorporate a true dynamic stretching condition to clarify its comparative influence. Investigating the time course of recovery following static stretching induced instability would also enhance evidence-based warm-up recommendations.

## Data Availability

The original contributions presented in the study are included in the article/supplementary material, further inquiries can be directed to the corresponding authors.

## References

[B1] Aytaç T. İşler A. K. (2025). Beyond the warm-up: understanding the post-activation performance enhancement. Spor Hekim. Derg. 60 (3), 114–121. 10.47447/tjsm.0879

[B2] Behm D. G. Chaouachi A. (2011). A review of the acute effects of static and dynamic stretching on performance. Eur. J. Appl. Physiol. 111 (11), 2633–2651. 10.1007/s00421-011-1879-2 21373870

[B3] Behm D. G. Blazevich A. J. Kay A. D. McHugh M. (2016). Acute effects of muscle stretching on physical performance, range of motion, and injury incidence in healthy active individuals: a systematic review. Appl. Physiol. Nutr. Metabolism 41 (1), 1–11. 10.1139/apnm-2015-0235 26642915

[B4] Behm D. G. Alizadeh S. Daneshjoo A. Hadjizadeh Anvar S. Graham A. Ali Z. (2023). Acute effects of various stretching techniques on range of motion: a systematic review with meta-analysis. Sports Medi. Open 9 (1), 107. 10.1186/s40798-023-00652-x 37962709 PMC10645614

[B5] Blazevich A. J. Babault N. (2019). Post-activation potentiation versus post-activation performance enhancement in humans: historical perspective, underlying mechanisms, and current issues. Front. Physiol. 10, 1359. 10.3389/fphys.2019.01359 31736781 PMC6838751

[B6] Bramah C. Mendiguchia J. Dos’ Santos T. Morin J.-B. (2024). Exploring the role of sprint biomechanics in hamstring strain injuries: a current opinion on existing concepts and evidence. Sports Med. 54 (4), 783–793. 10.1007/s40279-023-01925-x 37725240 PMC11052868

[B7] Budini F. Christova M. Gallasch E. Paul K. Rafolt D. Tilp M. (2018). Transient increase in cortical excitability following static stretching of plantar flexor muscles. Front. Physiol. 9, 530. 10.3389/fphys.2018.00530 29942261 PMC6004398

[B8] Chatzopoulos D. Galazoulas C. Patikas D. Kotzamanidis C. (2014). Acute effects of static and dynamic stretching on balance, agility, reaction time and movement time. J. Sports Sci. Med. 13 (2), 403–409. Available online at: https://pmc.ncbi.nlm.nih.gov/articles/PMC3990897/ . 24790497 PMC3990897

[B9] Denerel N. Ergün M. Yüksel O. Özgürbüz C. Karamızrak O. (2019). The acute effects of static and dynamic stretching exercises on dynamic balance performance. Spor Hekim. Derg. 54 (3), 148–157. 10.5152/tjsm.2019.127

[B10] Doyle K. P. Brinken C. M. Capito C. N. Karp E. T. Mariani M. N. Meza S. (2025). The impact of vertical jump height, hamstrings flexibility and strength on maximal sprint speed in division I track and field athletes. J. Strength Cond. Res. 39, 1023–1027. 10.1519/JSC.0000000000005175 40493661

[B11] Feng K. (2024). Literature review of the effects of static stretching and dynamic stretching on jumping performance in volleyball players. Front. Sport Res. 6 (4), 267–275. 10.25236/FSR.2024.060403

[B12] Guissard N. Duchateau J. (2004). Effect of static stretch training on neural and mechanical properties of the human plantar‐flexor muscles. Muscle Nerve Official J. Am. Assoc. Electrodiagn. Med. 29 (2), 248–255. 10.1002/mus.10549 14755490

[B13] Hindle K. B. Tyler J. W. Briggs W. O. Hong J. (2012). Proprioceptive neuromuscular facilitation (PNF): its mechanisms and effects on range of motion and muscular function. J. Human Kinetics 31, 105–113. 10.2478/v10078-012-0011-y PMC3588663 23487249

[B14] Hrvatin I. Puh U. (2021). Measurement properties of the numerical pain rating scale in patients with musculoskeletal impairments of the limbs-a systematic literature review.

[B15] Ingram L. A. Tomkinson G. R. d’Unienville N. M. A. Gower B. Gleadhill S. Boyle T. (2025). Mechanisms underlying range of motion improvements following acute and chronic static stretching: a systematic review, meta-analysis and multivariate meta-regression. Sports Med. Auckl. Nz 55 (6), 1449–1466. 10.1007/s40279-025-02204-7 PMC12152101 40180774

[B16] Koo T. K. Li M. Y. (2016). A guideline of selecting and reporting intraclass correlation coefficients for reliability research. J. Chiropractic Med. 15 (2), 155–163. 10.1016/j.jcm.2016.02.012 27330520 PMC4913118

[B17] Kranjc S. Fink M. Nakamura M. Kozinc Ž. (2025). Acute effects of proprioceptive neuromuscular facilitation stretching on rectus femoris muscle stiffness: a dose-response shear-wave elastography study. Front. Physiol. 15, 1496825. 10.3389/fphys.2024.1496825 39850447 PMC11754182

[B18] Lawry-Popelka B. Chung S. McCann R. S. (2022). Cross-education balance effects after unilateral rehabilitation in individuals with chronic ankle instability: a systematic review. J. Athl. Train. 57 (11-12), 1055–1061. 10.4085/1062-6050-625-21 36395371 PMC9875701

[B19] Lim K.-I Nam H.-C. Jung K.-S. (2014). Effects on hamstring muscle extensibility, muscle activity, and balance of different stretching techniques. J. Phys. Therapy Sci. 26 (2), 209–213. 10.1589/jpts.26.209 24648633 PMC3944290

[B20] Longo S. Cè E. Toninelli N. Esposito F. Coratella G. (2025). Muscle stretching: exploring the impact of different modalities on maximal range of motion and strength with practical recommendations. Sports Medicine-Open 11 (1), 126. 10.1186/s40798-025-00925-7 41201748 PMC12595194

[B21] Malek N. F. A. Nadzalan A.Md Tan K. Azmi A. M. N. Vasanthi R. K. Pavlović R. (2024). The acute effect of dynamic vs. proprioceptive neuromuscular facilitation stretching on sprint and jump performance. J. Funct. Morphol. Kinesiol. 9 (1), 42. 10.3390/jfmk9010042 38535422 PMC10971198

[B22] Patti A. Thomas E. Giustino V. Rossi C. Paoli A. Drid P. (2025). Effects of foam rolling and static stretching on ankle dorsiflexion and jumping ability: a randomized controlled trial. Biol. Sport 42 (4), 163–170. 10.5114/biolsport.2025.150042 41048241 PMC12490304

[B23] Plisky P. J. Rauh M. J. Kaminski T. W. Underwood F. B. (2006). Star excursion balance test as a predictor of lower extremity injury in high school basketball players. J. Orthopaedic Sports Phys. Therapy 36 (12), 911–919. 10.2519/jospt.2006.2244 17193868

[B24] Reiner M. Tilp M. Guilhem G. Morales-Artacho A. Nakamura M. Konrad A. (2021). Effects of a single proprioceptive neuromuscular facilitation stretching exercise with and without post-stretching activation on the muscle function and mechanical properties of the plantar flexor muscles. Front. Physiol. 12, 732654. 10.3389/fphys.2021.732654 34594241 PMC8476946

[B25] Rostami A. Majid Tabatabaeinejad S. Soltani M. Mirmoezzi M. (2025). Comparative effects of static stretching and PNF techniques on functional flexibility and postural balance in competitive taekwondo athletes. J. Sport Biomechanics 11 (2), 94–112. 10.61186/jsportbiomech.11.2.94

[B26] Saito A. Mizuno T. (2025). Effect of static stretching duration on modulation of H-reflex and tendon-reflex excitability of the soleus muscle in young men. Physiol. Rep. 13 (16), e70538. 10.14814/phy2.70538 40859631 PMC12381354

[B27] Simões E. Tavares N. Saraiva M. (2025). Acute effects of dynamic stretching on knee joint position sense and dynamic balance in recreational runners: a randomized controlled trial. Gait Posture 124, 110049. 10.1016/j.gaitpost.2025.110049 41242280

[B28] Takeuchi K. Nakamura M. Kakihana H. Tsukuda F. (2019). A survey of static and dynamic stretching protocol. Int. J. Sport Health Sci. 17, 72–79. 10.5432/ijshs.201829

[B29] Vieira D. C. L. Babault N. Hitier M. Durigan J. L. Q. Bottaro M. (2025). The acute effects of dynamic stretching on the neuromuscular system are independent of the velocity. Exp. Physiol. 110 (3), 494–505. 10.1113/EP092217 39763181 PMC11868028

[B30] Wang B. Wu B. Yang Y. Cai M. Li S. Peng H. (2024). Neuromuscular and balance adaptations following acute stretching exercise: a randomized control trial. Front. Physiol. 15, 1486901. 10.3389/fphys.2024.1486901 39691093 PMC11649666

[B31] Warneke K. Lohmann L. H. (2024). Revisiting the stretch-induced force deficit: a systematic review with multilevel meta-analysis of acute effects. J. Sport Health Sci. 13 (6), 805–819. 10.1016/j.jshs.2024.05.002 38735533 PMC11336295

[B32] Yan R. Lin G. Peng W. Chen Y. Sun P. Sun J. (2025). Time-dependent effects of acute stretching on power, balance, and flexibility in contemporary dancers: a randomized crossover trial. Sci. Rep. 15 (1), 15489. 10.1038/s41598-025-00027-0 40319037 PMC12049479

